# Effect of 4-Allyl-1-hydroxy-2-methoxybenzene (Eugenol) on Inflammatory and Apoptosis Processes in Dental Pulp Fibroblasts

**DOI:** 10.1155/2016/9371403

**Published:** 2016-12-04

**Authors:** Andrea Martínez-Herrera, Amaury Pozos-Guillén, Socorro Ruiz-Rodríguez, Arturo Garrocho-Rangel, Antonio Vértiz-Hernández, Diana María Escobar-García

**Affiliations:** ^1^Pediatric Dentistry Postgraduate Program, Faculty of Dentistry, San Luis Potosi University, 78290 San Luis Potosí, SLP, Mexico; ^2^Basic Sciences Laboratory, Faculty of Dentistry, San Luis Potosi University, 78290 San Luis Potosí, SLP, Mexico; ^3^Coordinación Académica Región Altiplano, San Luis Potosi University, 78700 Matehuala, SLP, Mexico

## Abstract

Eugenol (mixed with zinc oxide powder) is widely used as direct capping material during pulp therapy in primary teeth. The aim of the present study was to evaluate the effect of eugenol on diverse genes involved in inflammatory and cell apoptosis processes. The regulatory effect of eugenol on the expression of inflammation and apoptotic genes was evaluated in dental pulp fibroblasts from extracted third molars, cultured under concentration of eugenol of 13 *μ*M. Eugenol allowed the expression of inflammatory and apoptotic genes when compared with positive and negative controls. Eugenol is a proinflammatory agent when it is in direct contact with healthy tissues and behaves as an anti-inflammatory agent in tissues undergoing inflammatory/apoptotic processes, as in cases of pulp inflammation in primary teeth. These findings are relevant for dentistry, when considering the application of safer pulp treatments to grossly carious children's teeth.

## 1. Introduction

Dental caries is the most common chronic disease in childhood caused mainly by a poor, long-term oral hygiene. In its initial stages, the disease can be preventable and reversible; however, without appropriate and timely control, cariogenic bacteria may seriously destroy the hard dental tissues and consequently affect the dental pulp [1]. Dental pulp is a connective tissue composed mainly of fibroblasts and odontoblasts, with an inherent capability to produce reparative dentin in a favorable environment [2]. Hence, it is imperative to preserve the pulp vitality and function of primary teeth whenever possible.

In cases of pulp tissue inflammation by carious pulp exposure, there is a penetration of cariogenic Gram-positive and Gram-negative bacteria, the predominant microbiota in the oral cavity. It is well known that the cell wall structure of cariogenic bacteria is predominantly constituted of lipopolysaccharides (LPS). LPS can activate the pulp immune system, inducing the segregation of proinflammatory chemokines and cytokines, with an increase in capillary permeability [3]. Additionally, the pulp's role during the inflammatory response is to fight against infection and tissue damage through immune cells, such as fibroblasts and other cells that recognize damage from cariogenic bacteria [4]; fibroblasts activate the recognition of bacterial surface receptors, which initiates the inflammatory response by stimulating the production of cytokines, including proinflammatory, anti-inflammatory, and regulatory cytokines [5]. Furthermore, recruitment of neutrophils and polymorphonuclear cells occurs, which increases expression of the interleukins [6].

Maintaining the integrity and health of primary teeth in functional status until their natural exfoliation is one of the fundamental objectives of pediatric dentistry. It is essential for achieving normal oral function and facial growth [7]. For this purpose, pediatric dentistry practitioners employ diverse restorative or endodontic procedures for treating deep carious lesions with or without pulp tissue exposure in primary teeth. One of the most common and widely accepted clinical endodontic treatments in pediatric dentistry is the pulpotomy technique [8]. The objective of pulpotomy is to remove only the affected coronal pulp tissue, so that the unaffected radicular pulp tissue remains in place and functioning normally until the tooth is ready to exfoliate [9]. Because of its sedative or anodyne properties, low cost, simplicity, and efficacy, the zinc oxide/eugenol combination (ZOE) is frequently used as capping material over the radicular pulp when pulpotomies are performed in primary teeth [9].

Eugenol (4-allyl-1-hydroxy-2-methoxybenzene) is an essential oil extracted from the plant* Eugenia caryophyllata* [10], popularly known as “cloves,” which grows in different regions worldwide [11]. This oil has demonstrated to possess different biological properties, such as antimicrobial, analgesic, anti-inflammatory, antioxidant, antimutagenic, and anticarcinogenic effects [3, 12]. Recent* in vivo* studies have explored the potential anticancer property of eugenol, demonstrating the induction of apoptosis in several cancer cell lines from animal tumor models [4, 13]. On the other hand, through different experimental models, eugenol has proven to reduce Oxidative Stress (OS) by preventing oxidative damage [14–16]. Paradoxically, ZOE has also proven to possess harmful effects when placed directly on dentin tissue or when employed as pulpotomy material in primary teeth [17, 18]. Eugenol is cytotoxic for several types of human cell, including pulp fibroblasts; it reduces not only the growth and survival of these cells, but also their collagen synthesis and bone sialoprotein expression, which play a critical role in reparative dentine formation [18]. Therefore, it is necessary to obtain new information concerning why, when, and under which circumstances eugenol provides either beneficial or harmful effects in order to procure safer employment of ZOE in clinical pediatric dentistry when treating deeply carious primary teeth.

The aim of this study was to assess the anti-inflammatory effects of eugenol on cultured dental pulp fibroblasts, under inflammatory conditions. These effects were measured through the production of several inflammatory cytokines and the expression of inflammation related-genes.

## 2. Materials and Methods

### 2.1. Cell Cultures

The whole culture process was based on a previous reported method by Escobar-García et al. [19]. Fibroblasts of dental pulp were obtained from extracted caries-free third molars. The extracted molars were placed in a transport medium (Phosphate-Buffered Saline (PBS) solution with a 3% antibiotic mixture (1,000 U/mL Penicillin, 1 mg/mL Streptomycin, and Amphotericin B 2.5 mg/*μ*L)). Then, the pulp chamber was exposed with a diamond disc, and the pulp tissue was removed, washed with transport medium, and incubated in a collagenase solution (2 mg/mL) for 3 h at 37°C, 5% CO_2_, and 95% humidity. After centrifuging, the supernatant was removed. The remaining tissue was cultured for explant (tissue dissected into small, 1–3 mm pieces), together with 3 mL of Dulbecco's Modified Eagle Medium (DMEM) culture medium enriched with 10% Fetal Bovine Serum (FBS) and an antibiotic combination (1,000 U/mL Penicillin, 1 mg/mL Streptomycin, and Amphotericin B 2.5 mg/*μ*L); the mixture was incubated at 37°C, 5% CO_2_, and 95% humidity, and the culture medium was replaced every other day.

### 2.2. Treatment and Control Groups

Once the cell cultures reached a confluence of >70%, fibroblasts were assigned to different study groups. Five groups were designated as follows: (1) fibroblasts placed in an enriched FBS culture medium (negative control group); (2) fibroblasts treated only with LPS 10 *μ*g/mL (lipopolysaccharide from* Escherichia coli* 0127:B8), without eugenol (positive control of inflammation) for 48 h; (3) fibroblasts treated for 24 h with LPS 10 *μ*g/mL, and eugenol 13 *μ*M for 24 h (experimental group A); (4) fibroblasts treated with eugenol for 24 h, without LPS (experimental group B); and (5) fibroblasts treated only with H_2_O_2_ for 24 h, without eugenol (positive control of cell apoptosis). In experimental groups (A and B), the medium was modified according to the concentration of eugenol of 13 *μ*M (this concentration is based on previous work reported by Escobar-García et al. [19]) and then incubated for an additional 24 h. Each experiment was carried out in triplicate. After the experiments were conducted, cells were detached from the culture box employing trypsin ethylenediaminetetraacetic acid (EDTA) 0.025% and collected for subsequent RNA isolation.

### 2.3. RNA Isolation

RNA was isolated from cultured fibroblasts through the method of Tri Reagent (Sigma-Aldrich BioSciences, St. Louis, MO, USA) following the manufacturer's instructions. The extracted RNA was then suspended in RNase-free water. The concentration of genetic material was quantified using the NanoDrop reagent (Thermo Scientific FC Multiskan®; Thermo Scientific, Vantaa, Finland).

### 2.4. cDNA Synthesis

Complementary DNA (cDNA) synthesis was performed with 1 *μ*g of RNA utilizing the reverse transcription long range reverse transcriptase (QIAGEN GmbH, D-40724 Hilden, Germany) reagent and was incubated twice, that is, for 60 min at 37°C and for 5 min at 85°C in order to inactivate the transcriptase.

### 2.5. Amplification of the PCR Products

Specific amplification for inflammation (NF-*κ*B, IL-1*β*, TNF-*α*, and VEGFA) and apoptotic (p53 and Apaf-1) genes was performed separately utilizing primer pairs under the amplifications (Table 1). Forward and reverse PCR reaction was carried out in 25 *μ*L of green GoTaq (Promega Co., Madison, WI, USA) Master Mix, consisting of cDNA 250 ng and primer 0.5 *μ*M. For designing the primers, the Amplifix 1.5.4 program was employment; alignment and temperatures were confirmed using Oligocalculator online software. The sequences of the primers and the annealing temperatures are described in Table 1. Finally, the PCR products underwent electrophoresis on agarose gels 1%, 90 volts for 1 h. The agarose gels were analyzed in a Quantity One BioRad (BioRad Laboratories, Hercules, CA, USA) Photo Register, in which the relative expression of each gene was compared with the control group; glyceraldehyde 3-phosphate dehydrogenase (GAPDH) was utilized as housekeeping gene.

### 2.6. Statistical Analyses

Results from all study groups were continuous variables and are therefore expressed as means and standard deviations (SD): three data sets per control group and six data sets from each treatment group. Means were compared by the one-way analysis of variance (ANOVA) followed by Tukey test for multiple comparisons, employing SigmaPlot ver. 11.0 statistical software (Systat Software, Inc.). A *p* value of <0.05 was taken as statistically significant.

## 3. Results

### 3.1. Eugenol and Inflammation

**Figure 1:**
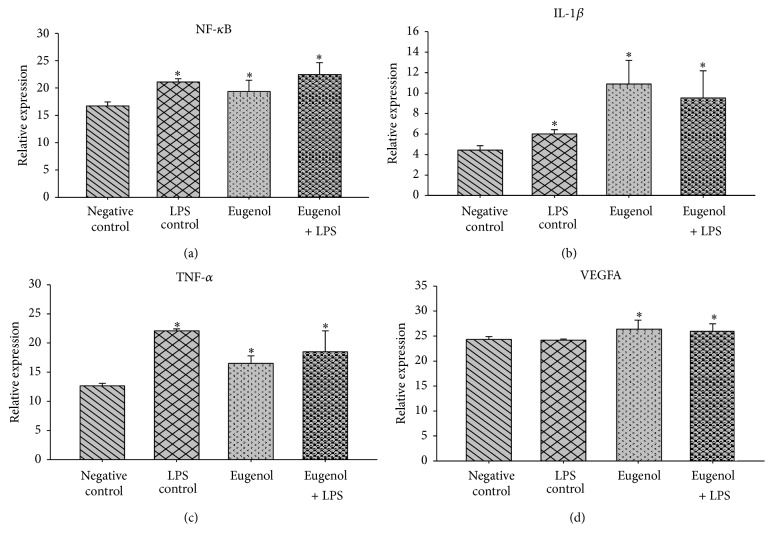
Relative expression of genes involved in the inflammatory process. LPS-lipopolysaccharides; NF-*κ*B-nuclear factor kappa B; IL-1*β*-interleukin 1 beta; TNF-*α*-tumor necrosis factor-alfa; VEGFA-vascular endothelial growth factor A. ^*∗*^The differences in the mean values among the treatment groups with respect to negative control are greater than what would be expected by chance; there is a statistically significant difference (*p* < 0.05).

To assess whether there were differences among fibroblast gene expressions, comparisons were made as follows: (a) negative control versus cell groups treated with eugenol and (b) positive control (LPS) versus cell groups treated with eugenol. The first comparison (negative control versus cell groups treated with eugenol) demonstrated that nuclear factor kappa B (NF-*κ*B) is expressed 1.16-times more than the negative control, corresponding to a 16% increase (Figure 1(a)). Interleukin 1 beta (IL1-*β*) is expressed 2.4-fold above the control corresponding to an increase in expression of 145% (Figure 1(b)). Tumor necrosis factor alpha (TNF-*α*) is expressed 1.3 times that of control cells, representing a 30% increase in expression (Figure 1(c)). The treated cells of the vascular endothelial growth factor A (VEGFA) gene expressed eugenol at 1.08 times the negative control, representing a 6.5% increase in gene expression (Figure 1(d)). By performing a second analysis in which the expression was compared to inflammatory genes in eugenol-treated cells treated versus cells that had previously undergone treatment with LPS at a concentration of 10 *μ*g/mL, the NF-*κ*B gene was expressed 0.9 times that of the control, representing 8.5% inhibition in expression (Figure 1(a)). TNF-*α* is expressed only 0.7-fold, representing 25.4% inhibition (Figure 1(b)), while IL1-*β* and VEGFA continued to exhibit proinflammatory behavior, expressed as 1.8-fold and 1.08-fold that of control inflammation, respectively, corresponding to 81.2% IL1-*β* and 7.2% of VEGFA induction in increased expression (Figures 1(c) and 1(d)).

### 3.2. Effect of Eugenol on the Apoptosis Process

**Figure 2:**
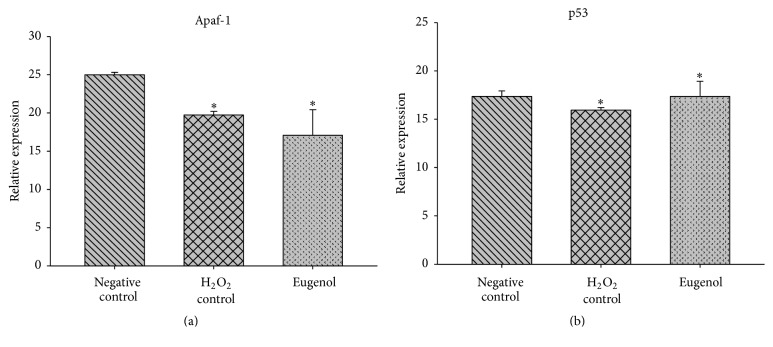
Relative expression of genes involved in the apoptotic process. Apaf-1-apoptotic peptidase activation factor 1; p53-tumor suppressor p53. ^*∗*^The differences in the mean values among the treatment groups with respect to negative control are greater than what would be expected by chance; there is a statistically significant difference (*p* < 0.05).

Two genes and their expressions were involved in the apoptosis process: tumor suppressor p53 (p53) and the apoptotic peptidase activation factor 1 (Apaf-1). The p53 gene was expressed 1.04 times that of the control or a 4% increase in induction (Figure 2(a)), which is statistically significant (*p* < 0.05). Likewise, Apaf-1 gene-treated cells inhibited eugenol expression in 32% (0.68 times) with regard to the control (Figure 2(b)); this inhibition was also significant (*p* < 0.05).

## 4. Discussion

Premature loss of primary teeth can lead to malocclusion and phonetic or functional problems in the growing child. Therefore, it is necessary that pediatric dentistry practitioners always intend to preserve primary tooth pulp vitality free of inflammation/infection [20]. Pathogenic bacteria and their products are involved in severe forms of inflammation when the pulp is exposed by caries. However, it has been proven that pulpal inflammation occurs even in the absence of bacteria and that other factors participate in the inflammatory process; for example, considering that ZOE-based materials release small amounts of eugenol when placed in contact with dentin tissue, eugenol can diffuse through dentinal tubules, causing potential injury to the pulp [21]. After mixing ZOE, a chelation reaction occurs and zinc eugenolate is formed. This compound is readily hydrolyzed when exposed to dentinal tubule fluids to yield eugenol and zinc hydroxide; free eugenol can diffuse through the tubules up to reaching the pulp tissue and its cell components, such as fibroblasts [17, 21, 22].

**Figure 3:**
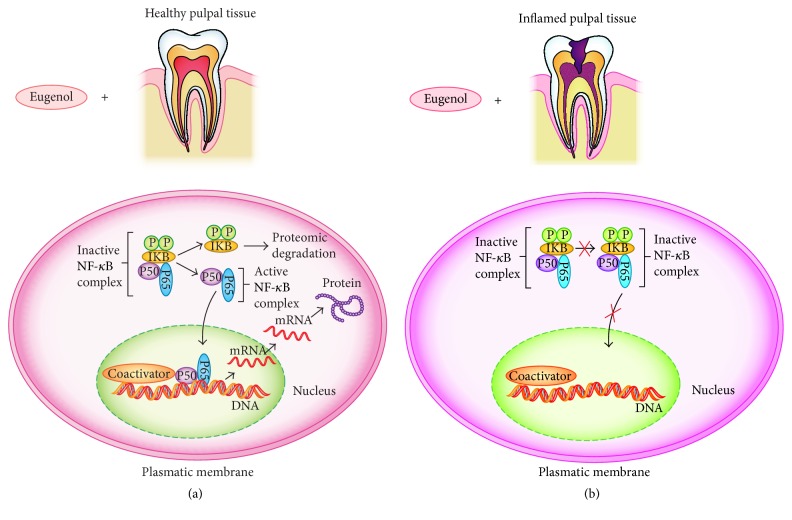
A possible translocation of NF-*κ*B for healthy pulp tissue (a) and inflamed pulpal tissue (b). (a) Healthy pulpal tissue. Eugenol allowed interaction with the IKB complex that mediates phosphorylation and degradation of inhibitor protein IKB, in turn activating the NF-*κ*B transcription-B factor and translocation of NF-*κ*B into the nucleus, where it participates in the activation of other genes involved in both inflammation and apoptosis. (b) Inflamed pulpal tissue. NF-*κ*B expression is inhibited when there is preexisting inflammation because they do not carry out IKB phosphorylation inhibitor activities. P-phosphorylation; IKB-complex NF-*κ*B inhibitor; P50-homodimer P50; P65-homodimer P65; NF-*κ*B-nuclear factor kappa B.

The present results strongly suggest that eugenol induces the expression of diverse genes involved in the inflammatory process; it was observed here that gene expression was significantly increased when fibroblasts were treated with eugenol as compared with untreated fibroblasts (Figure 3(a)). However, eugenol inhibited the expression of NF-*κ*B when the preexisting inflammatory-inducted process was present (e.g., with LPS), in this case, the preexistence of inflammation, such as the real condition of the dental pulp in primary teeth (Figure 3(b)). Eugenol allowed interaction with the NF-*κ*B complex inhibitor (IKB); the latter mediates protein IKB phosphorylation and degradation, in turn activating the NF-*κ*B transcription factor to promote nonapoptotic signaling pathways and gene expression via antiapoptotic pathways, ensuring nonapoptotic signaling activation [23]. Furthermore, eugenol has been considered as anti-inflammatory agent due to its well-known participation in the inhibition of cyclooxygenase 2 (COX2) expression. This protein is responsible for the production of inflammatory prostaglandins. Upregulation of COX2 is also associated with increased cell adhesion, phenotypic changes, resistance to apoptosis, and tumor angiogenesis [24, 25].

Fibroblast cultures constitute an adequate model for studying the mechanisms of inflammation and cell apoptosis at a molecular level [26]. It has been reported that NF-*κ*B comprises a transcription factor regulating the inflammatory process and participating in the transcription of many cytokines, such as TNF-*α*, IL1-*β*, and IL-6 [27]; thus, the inhibitors of this factor can be considered anti-inflammatory agents [28]. This gene also plays a significant role in other processes, including cell development, growth, and proliferation [29]. TNF-*α* is an important proinflammatory cytokine secreted by many cell types, such as macrophages, lymphocytes, fibroblasts, and keratocytes, in response to an inflammatory reaction, infection, or environmental changes [30]. Within the same context, IL1-*β* is an important mediator of the inflammatory response as a proinflammatory cytosine, involved in different cellular activities including proliferation, differentiation, and apoptosis [31]; it acts during the acute response phase in antimicrobial defense. VEGFA is a growth factor expressed primarily in endothelial cells, with various effects, such as mediating increased vascular permeability, angiogenesis promotion, cell migration, and apoptosis inhibition [32].

Apoptosis or programmed cell death is a complex phenomenon comprising the delicate regulation of signaling proteins via gene expression and/or protein activity. Apoptosis participates in various physiological events involved in many diseases, including cancer and neurodegenerative disorders [33, 34]. The process can be initiated intrinsically or extrinsically, depending on the nature of the cell death signal. After receiving intrinsic apoptotic stimuli, several proapoptotic proteins are released from the mitochondria into the cytosol, involving a large number of genes, such as p53 and Apaf-1 [23]. p53 is a protein encoding a tumor suppressive gene; thus, the encoded protein responds to diverse stressing mechanisms in the cell, which regulates the expression of target genes and induces cell cycle arrest, apoptosis, senescence, DNA repair, and changes in cell metabolism [35]. Apaf-1 is a gene encoding a cytoplasmic protein, which promotes the apoptosis process, mediates cytochrome C breaking, and activates procaspase 9; this process finally activates caspase 3, responsible for the onset of apoptosis [36].

NF-*κ*B is a heterodimer complex that binds to kappa-B DNA sites of their target genes. Individual dimers have preferences for different kappa-B sites, to which they can bind with distinguishable affinity and specificity. This dimer complex is localized in the cytoplasm in an inactive state bonded to the kappa-B inhibitor (I-kappa-B). Activation conventionally occurs by phosphorylation of I-kappa-through the I-kappa-B kinase (IKK), in response to various activators, and is subsequently degraded, thereby releasing the B-complex; once activated, NF-*κ*B is then translocated into the nucleus. This gene participates in the regulation of >500 genes involved in some inflammatory process, cell survival, proliferation, angiogenesis, and metastasis [37]. Because NF-*κ*B is a regulator of the inflammatory process, inhibition of its expression is considered an anti-inflammatory effect [23].

Apaf-1 is a cytoplasmic protein and one of the axes controlling the apoptosis regulatory network. Eugenol is able to significantly suppress the expression of Apaf-1, suggesting that eugenol does not yield a programmed cell death, although it is able to significantly induce the expression of p53, which may indicate that the cell is in contact with this oil. If there are any alterations in the layers, a genome-increased expression of p53 is motivated, in order to repair damage to genetic material, but without achieving the final apoptosis process. Therefore, apoptosis is a normal process of many multicellular organisms; however, alterations in this process are associated with the pathogenesis of different diseases.

## 5. Conclusions

According to the present study's results, low-concentrated eugenol possesses the property of an anti-inflammatory agent when the pulp tissue is found in an inflamed state, as is the case of reversible pulpitis in primary teeth, due to being capable of inhibiting gene expression, such as that of NF-*κ*B and TNF-*α*.

## Figures and Tables

**Table 1:** Primers and conditions employed for evaluation of gene expression.

Gene symbol	Primer sequence (5′-3′)	Annealing temperature (°C)	Size of PCR product (bp)
Apaf-1	Fw: ATGAGGCTCTAGACGAAGCCATGT	55.7	490
	Rv: TGAATCAGATGAGCAGGGCCTACA		

P53	Fw: TTGCTATCTGGGACAGCCAAGT	59.1	491
	Rv: CAGGCACAAACATGCACCTCAAAG		

TNF-*α*	Fw: CGTCTCCTACCAGACCAAGGTCAA	58.8	401
	Rv: TTCCTGAATCCCAGGTTTCGAAGTG		

NF-*κ*B	Fw: CCTTGCACTTGGCAGTGATCACTA	59.3	294
	Rv: ACTTCTGCTCCTGAGCATTGACG		

VEGFA	Fw: ATCACCGAGCCCGGAAGATTAGA	55	384
	Rv: CGGTGTTCCCAAAACTGGGTCAT		

GAPDH	Fw: CCATCAATGACCCCTTCATTGACC	65.2	435
	Rv: TGGTCATGAGTCCTTCCACGAT		

IL1-*β*	Fw: CGATGCACCTGTACGATCACTGAA	56.9	223
	Rv: CAACACGCAGGACAGGTACAGATT		

Apaf-1-apoptotic peptidase activation factor 1; P53-tumor suppressor P53; TNF-*α*-tumor necrosis factor-alfa; NF-*κ*B-nuclear factor kappa B; VEGFA-vascular endothelial growth factor A; GAPDH-glyceraldehyde 3-phosphate dehydrogenase; IL-1*β*-interleukin 1 beta.

## References

[1] González-Lara A., Ruiz-Rodríguez M. S., Pierdant-Pérez M., Garrocho-Rangel J. A., Pozos-Guillén A. J. (2016). Zinc Oxide–eugenol pulpotomy in primary teeth: a 24-month follow-up. *Journal of Clinical Pediatric Dentistry*.

[2] Garrocho-Rangel A., Flores H., Silva-Herzog D., Hernandez-Sierra F., Mandeville P., Pozos-Guillen A. J. (2009). Efficacy of EMD versus calcium hydroxide in direct pulp capping of primary molars: a randomized controlled clinical trial. *Oral Surgery, Oral Medicine, Oral Pathology, Oral Radiology and Endodontology*.

[3] Laughlin R. C., Mickum M., Rowin K., Adams L. G., Alaniz R. C. (2015). Altered host immune responses to membrane vesicles from Salmonella and Gram-negative pathogens. *Vaccine*.

[4] Newton K., Dixit V. M. (2012). Signaling in innate immunity and inflammation. *Cold Spring Harbor Perspectives in Biology*.

[5] Bachiega T. F., De Sousa J. P. B., Bastos J. K., Sforcin J. M. (2012). Clove and eugenol in noncytotoxic concentrations exert immunomodulatory/ anti-inflammatory action on cytokine production by murine macrophages. *The Journal of Pharmacy and Pharmacology*.

[6] Modaresi J., Davoudi A., Badrian H., Sabzian R. (2016). Irreversible pulpitis and achieving profound anesthesia: complexities and managements. *Anesthesia: Essays and Researches*.

[7] Knight G. M., McIntyre J. M., Craig G. G., Mulyani, Zilm P. S., Gully N. J. (2007). An in vitro investigation of marginal dentine caries abutting composite resin and glass ionomer cement restorations. *Australian Dental Journal*.

[8] Parisay I., Ghoddusi J., Forghani M. (2015). A review on vital pulp therapy in primary teeth. *Iranian Endodontic Journal*.

[9] Fuks A., Perets B. (2016). *Pediatric Endodontics—Current Concepts in Pulp Therapy for Primary and Young Permanent Teeth*.

[10] Daniel A. N., Sartoretto S. M., Schmidt G., Caparroz-Assef S. M., Bersani-Amado C. A., Cuman R. K. N. (2009). Anti-inflammatory and antinociceptive activities of eugenol essential oil in experimental animal models. *Revista Brasileira de Farmacognosia*.

[11] Agra M. D. F., Silva K. N., Basílio I. J. L. D., De Freitas P. F., Barbosa-Filho J. M. (2008). Survey of medicinal plants used in the region Northeast of Brazil. *Brazilian Journal of Pharmacognosy*.

[12] Estevão-Silva C. F., Kummer R., Fachini-Queiroz F. C. (2014). Anethole and eugenol reduce in vitro and in vivo leukocyte migration induced by fMLP, LTB4, and carrageenan. *Journal of Natural Medicines*.

[13] Sarkar A., Bhattacharjee S., Mandal D. P. (2015). Induction of apoptosis by eugenol and capsaicin in human gastric cancer AGS cells - elucidating the role of p53. *Asian Pacific Journal of Cancer Prevention*.

[14] Morsy M. A., Fouad A. A. (2008). Mechanisms of gastroprotective effect of eugenol in indomethacin-induced ulcer in rats. *Phytotherapy Research*.

[15] Nagababu E., Lakshmaiah N. (1992). Inhibitory effect of eugenol on non-enzymatic lipid peroxidation in rat liver mitochondria. *Biochemical Pharmacology*.

[16] Kabuto H., Tada M., Kohno M. (2007). Eugenol [2-methoxy-4-(2-propenyl)phenol] prevents 6-hydroxydopamine-induced dopamine depression and lipid peroxidation inductivity in mouse striatum. *Biological & Pharmaceutical Bulletin*.

[17] Hume W. R. (1986). The pharmacologic and toxicological properties of zinc oxide-eugenol. *The Journal of the American Dental Association*.

[18] Anpo M., Shirayama K., Tsutsui T. (2011). Cytotoxic effect of eugenol on the expression of molecular markers related to the osteogenic differentiation of human dental pulp cells. *Odontology*.

[19] Escobar-García M., Rodríguez-Contreras K., Ruiz-Rodríguez S., Pierdant-Pérez M., Cerda-Cristerna B., Pozos-Guillén A. (2016). Eugenol toxicity in human dental pulp fibroblasts of primary teeth. *Journal of Clinical Pediatric Dentistry*.

[20] Koshy S., Love R. M. (2004). Endodontic treatment in the primary dentition. *Australian Endodontic Journal*.

[21] De Souza Costa C. A., Hebling J., Scheffel D. L. S., Soares D. G. S., Basso F. G., Ribeiro A. P. D. (2014). Methods to evaluate and strategies to improve the biocompatibility of dental materials and operative techniques. *Dental Materials*.

[22] Markowitz K., Moynihan M., Liu M., Kim S. (1992). Biologic properties of eugenol and zinc oxide-eugenol. A clinically oriented review. *Oral Surgery, Oral Medicine, Oral Pathology*.

[23] Chhibber-Goel J., Coleman-Vaughan C., Agrawal V. (2016). *γ*-Secretase activity is required for regulated intramembrane proteolysis of tumor necrosis factor (TNF) receptor 1 and TNF-mediated pro-apoptotic signaling. *The Journal of Biological Chemistry*.

[24] Murakami Y., Kawata A., Seki Y. (2012). Comparative inhibitory effects of magnolol, honokiol, eugenol and bis-eugenol on cyclooxygenase-2 expression and nuclear factor-kappa B activation in RAW264.7 macrophage-like cells stimulated with fimbriae of *Porphyromonas gingivalis*. *In Vivo*.

[25] Hassan L., Pinon A., Limami Y. (2016). Resistance to ursolic acid-induced apoptosis through involvement of melanogenesis and COX-2/PGE2 pathways in human M4Beu melanoma cancer cells. *Experimental Cell Research*.

[26] Pereira J. F., Awatade N. T., Loureiro C. A., Matos P., Amaral M. D., Jordan P. (2016). The third dimension: new developments in cell culture models for colorectal research. *Cellular and Molecular Life Sciences*.

[27] Yang R., Yang L., Shen X. (2012). Suppression of NF-*κ*B pathway by crocetin contributes to attenuation of lipopolysaccharide-induced acute lung injury in mice. *European Journal of Pharmacology*.

[28] Di R., Huang M.-T., Ho C.-T. (2011). Anti-inflammatory activities of mogrosides from Momordica grosvenori in murine macrophages and a murine ear edema model. *Journal of Agricultural and Food Chemistry*.

[29] Huang X., Liu Y., Lu Y., Ma C. (2015). Anti-inflammatory effects of eugenol on lipopolysaccharide-induced inflammatory reaction in acute lung injury via regulating inflammation and redox status. *International Immunopharmacology*.

[30] Baud V., Karin M. (2001). Signal transduction by tumor necrosis factor and its relatives. *Trends in Cell Biology*.

[31] Piccioli P., Rubartelli A. (2013). The secretion of IL-1*β* and options for release. *Seminars in Immunology*.

[32] Lee M.-S., Ghim J., Kim S.-J. (2015). Functional interaction between CTGF and FPRL1 regulates VEGF-A-induced angiogenesis. *Cellular Signalling*.

[33] Yun T., Yu K., Yang S. (2016). Acetylation of p53 protein at lysine 120 up-regulates Apaf-1 protein and sensitizes the mitochondrial apoptotic pathway. *The Journal of Biological Chemistry*.

[34] Franco R., Cidlowski J. A. (2009). Apoptosis and glutathione: beyond an antioxidant. *Cell Death and Differentiation*.

[35] Vijayakumaran R., Tan K. H., Miranda P. J., Haupt S., Haupt Y. (2015). Regulation of mutant p53 protein expression. *Frontiers in Oncology*.

[36] Koh D.-I., An H., Kim M.-Y. (2015). Transcriptional activation of APAF1 by KAISO (ZBTB33) and p53 is attenuated by RelA/p65. *Biochimica et Biophysica Acta—Gene Regulatory Mechanisms*.

[37] Sung B., Prasad S., Yadav V. R., Aggarwal B. B. (2012). Cancer cell signaling pathways targeted by spice-derived nutraceuticals. *Nutrition and Cancer*.

